# Effect of Antidiabetic Drugs on Bone Health in Patients with Normal Renal Function and in Chronic Kidney Disease (CKD): Insight into Clinical Challenges in the Treatment of Type 2 Diabetes

**DOI:** 10.3390/jcm12237260

**Published:** 2023-11-23

**Authors:** Cristiana Cipriani, Gabriella Lauriero, Giovanni Tripepi, Serge Ferrari, Jordi Bover, Maura Ravera, Simona Barbuto, Giuseppe Cianciolo, Luca De Nicola, Maria Luisa Brandi, Salvatore Minisola, Maria Cristina Mereu, Giovanni Corrao, Lucia Del Vecchio, Maria Fusaro

**Affiliations:** 1 Department of Clinical, Internal, Anesthesiological and Cardiovascular Sciences, Sapienza University of Rome, 00161 Rome, Italy; salvatore.minisola@uniroma1.it; 2 Nephrology and Dialysis Unit, Ospedale “F. Perinei”, ASL of Bari, 70022 Bari, Italy; gabriella.lauriero@asl.bari.it; 3 National Research Council (CNR), Institute of Clinical Physiology, Section of Biostatistics, 89124 Reggio Calabria, Italy; giovanniluigi.tripepi@cnr.it; 4 Department of Medicine, Service of Bone Diseases, Geneva University Hospital and Faculty of Medicine, 1205 Geneva, Switzerland; serge.ferrari@unige.ch; 5 Department of Nephrology, University Hospital Germans Trias i Pujol, 08916 Badalona, Spain; jordicatalonio@yahoo.es; 6 REMAR-IGTP Group, Research Institute Germans Trias i Pujol, Can Ruti Campus, 08916 Badalona, Spain; 7 Nephrology, Dialysis, and Transplantation, University of Genoa and Policlinico San Martino, 16132 Genoa, Italy; maura.ravera@hsanmartino.it; 8 Nephrology, Dialysis and Renal Transplant Unit, IRCCS, Azienda Ospedaliero-Universitaria di Bologna, Alma Mater Studiorum University of Bologna, 40126 Bologna, Italy; simona.barbuto@studio.unibo.it (S.B.); giuseppe.cianciolo@aosp.bo.it (G.C.); 9 Division of Nephrology, University of Campania “Luigi Vanvitelli”, 80137 Naples, Italy; luca.denicola@unicampania.it; 10 Fondazione Italiana Ricerca sulle Malattie dell’Osso (FIRMO Onlus), 50129 Florence, Italy; marialuisa.brandi@unifi.it; 11 Independent Researcher, 09100 Cagliari, Italy; crissmer5412@gmail.com; 12 Unit of Biostatistics, Epidemiology and Public Health, Department of Statistics and Quantitative Methods, University of Milano-Bicocca, 20126 Milan, Italy; giovanni.corrao@unimib.it; 13 Department of Nephrology and Dialysis, Sant’ Anna Hospital, ASST Lariana, 22042 Como, Italy; lucia.delvecchio@asst-lariana.it; 14 National Research Council (CNR), Institute of Clinical Physiology, 56124 Pisa, Italy; 15 Department of Medicine, University of Padua, 35128 Padua, Italy

**Keywords:** type 2 diabetes, antidiabetics, chronic kidney disease, bone, fracture, diabetic kidney disease

## Abstract

Among the metabolic changes occurring during the course of type 2 diabetes (T2DM) and diabetic kidney disease (DKD), impaired bone health with consequent increased fracture risk is one of the most complex and multifactorial complications. In subjects with diabetic kidney disease, skeletal abnormalities may develop as a consequence of both conditions. In the attempt to define a holistic approach to diabetes, potential effects of various classes of antidiabetic drugs on the skeleton should be considered in the setting of normal kidney function and in DKD. We reviewed the main evidence on these specific topics. Experimental studies reported potential beneficial and harmful effects on bone by different antidiabetics, with few data available in DKD. Clinical studies specifically designed to evaluate skeletal effects of antidiabetics have not been performed; notwithstanding, data gleaned from randomized controlled trials and intervention studies did not completely confirm observations made by basic research. In the aggregate, evidence from meta-analyses of these studies suggests potential positive effects on fracture risk by metformin and glucagon-like peptide-1 receptor agonists, neutral effects by dipeptidyl peptidase-4 inhibitors, sodium–glucose cotransporter-2 inhibitors, and sulfonylureas, and negative effects by insulin and thiazolidinediones. As no clinical recommendations on the management of antidiabetic drugs currently include fracture risk assessment among the main goal of therapy, we propose an integrated approach with the aim of defining a patient-centered management of diabetes in chronic kidney disease (CKD) and non-CKD patients. Future clinical evidence on the skeletal effects of antidiabetics will help in optimizing the approach to a personalized and more effective therapy of diabetes.

## 1. Introduction

The complex interaction between bone and glucose metabolism involves several organs (adipose tissue, bone, muscle, bone marrow adipose tissue, gastrointestinal (GI) tract, vessels, and kidneys), molecules (advanced glycation end-products, insulin, osteocalcin, sclerostin, bone morphogenetic proteins (BMPs), etc.), and signaling pathways (Wnt signaling, receptor activator of nuclear factor-kappa B ligand (RANKL), osteoprotegerin (OPG), etc.). Taken together, all these mechanisms are responsible for a mutual and dynamic interplay between the two systems, whose clinical implications range from increased fracture risk in patients with diabetes to the significant skeletal effect of antidiabetic drugs, as well as potential action of bone-active agents on glucose metabolism. Hence, the system is even more complicated in patients with diabetes and chronic kidney disease (CKD). 

Treatment of diabetes has extensively changed in the last two decades. The most recent national and international guidelines advocate for a personalized management of patients in which, alongside the central role of glucose control, treatment of comorbidities has utmost importance [1]. In particular, cardiovascular and renal disorders may be successfully targeted by specific treatments [1]. With reference to skeletal fragility, the most recent guidelines include detailed recommendations on how to screen patients by the use of tools available in clinical practice with specific indications for diabetes [2]. In terms of treatment, studies demonstrated that antiresorptives increase femoral bone mineral density (BMD) and reduce fracture risk to a similar extent in subjects with type 2 diabetes (T2DM) compared to the non-diabetic patients [3]. Post hoc analyses on the use of bone-forming agents, teriparatide and abaloparatide, reported significant antifracture efficacy in diabetic patients [4,5]. Similar analyses were not specifically performed in CKD patients with T2DM and increased fracture risk. Additionally, there have been reports of potential positive and detrimental effects of antidiabetic medications on bone metabolism [6,7]. Notwithstanding, no recommendations are currently available on how to manage antidiabetic therapy with the aim of targeting fracture risk reduction in both T2DM patients with and without CKD. We narratively reviewed the most updated literature on the skeletal effects of antidiabetic drugs in these two populations with the aim of addressing these issues and outlined potential relevant clinical applications of the findings.

## 2. Search Strategy

We searched the terms “diabetes”, “type 2 diabetes”, “antidiabetics”, “bone”, “fracture”, “bone mineral density”, “bone quality”, “chronic kidney disease”, and “diabetic kidney disease” on PubMed and retrieved peer-reviewed articles published from 2013 to 2023. We retrieved personal files and references to identify relevant articles published before 2013. We reviewed the articles and cited the most relevant ones published in English. As far as clinical studies are concerned, we prioritized randomized controlled trials, systematic reviews, and meta-analyses. Finally, we retrieved epidemiological data from the International Diabetes federation, World Health Organization, and United States Renal Data System websites.

## 3. Epidemiology and Pathophysiology of Type 2 Diabetes and Diabetic Kidney Disease

Diabetes mellitus is one of the fastest growing global health emergencies. Currently, 537 million adults aged 20–79 are living with diabetes worldwide; this number will likely increase to 643 million by 2030 and 783 million by 2045 [8]. According to the International Diabetes Federation (IDF), diabetes caused 6.7 million deaths and was responsible of at least USD 966 billion of health expenditure in 2021 [8]. 

Chronic kidney disease is a common complication in subjects with diabetes [9]. In the United States, CKD is diagnosed in more than 25% of diabetic patients; clinical evidence estimated that roughly 39% of subjects with diabetes develop CKD during their lifetime [10]. Studies have shown that the prevalence of diabetes increased from 2003–2006 to 2015–2018 to a figure of 9.7% among individuals without CKD and 32.8% in those with CKD [11].

Chronic kidney disease is defined as an elevated urine albumin excretion (albumin-to-creatinine ratio ≥ 30 mg/g) or reduced glomerular filtration rate (GFR < 60 mL/min/1.73 m^2^), or both [12,13]. The clinical, social, and economic impact of diabetic kidney disease (DKD) should be considered not only in light of the risk of progression to end-stage kidney disease but also in terms of increased cardiovascular risk [14]. 

In terms of pathophysiology, it is well known that risk factors for T2DM include a combination of non-modifiable (ethnicity, family history/genetic predisposition) and modifiable risk factors (obesity, low physical activity, and an unhealthy diet) [15]. Most patients with T2DM are obese or have increased body fat, with a predominant distribution in the abdominal region; the adipose tissue contributes to insulin resistance through a number of inflammatory processes, such as an increased release of free fatty acid and adipokine deregulation [15]. The resulting chronic inflammatory state represents the hallmark in the pathogenesis of T2DM [15]. Insulin resistance contributes to increased glucose production in the liver and decreased glucose uptake in the muscle, liver, and adipose tissue [15]. The combination of insulin resistance and an inadequate compensatory insulin response may progressively lead to β-cell dysfunction and relative insulin deficiency [15]. Insulin secretion is decreased, and the maintenance of adequate glucose levels is further impaired with a consequent worsening of complications [15]. Heterogeneity of the pathophysiology of T2DM implies perturbation of other signaling pathways in several systems and target tissues. Among them, the immune system may be involved in the development of autoimmune diabetes; aging and cell senescence in β cells might play a role in the pathogenesis of age-related diabetes [16].

With reference to DKD, there are three determinant processes typically described. Glomerular hypertrophy leading to hyperfiltration is present in roughly 40% of patients with type 2 diabetes as an early manifestation of DKD [13,17]. Glomerular and tubulointerstitial inflammation is another key mechanism associated with the activation of different chemokines, cytokines, and profibrotic factors [13]. Finally, there is dysregulation in cellular apoptosis and changes in the extracellular matrix [13]. Taken together, all these mechanisms lead to the thickening of the glomerular basal membrane, podocyte depletion, mesangial matrix expansion, and tubular damage, with consequent vascular remodeling, endothelial dysfunction, glomerulosclerosis, and tubulointerstitial fibrosis [13,18,19]. As a consequence, microalbuminuria (30–300 mg of albumin excreted per day) and, in the latest stages, macroalbuminuria, develop (>300 mg of albumin per day or even nephrotic range proteinuria: albuminuria > 2.200 mg/day) [12,20]. 

## 4. Bone Health in Type 2 Diabetes and CKD

In T2DM, the increased fracture risk is seen in the setting of normal–high normal BMD. Conversely, bone quality is impaired at the cortical sites [21]. Trabecular microarchitecture, as assessed by trabecular bone score (TBS), is significantly reduced in T2DM compared to the reference population, and associated with elevated fracture risk [21]. The use of TBS is recommended, when available, in the fracture risk assessment of T2DM subjects with the aim of better delineating the risk profile in the setting of normal BMD [21]. A recent meta-analysis of 12 studies assessing bone quality using high-resolution peripheral quantitative computed tomography (HR-pQCT) in a total of 516 patients with T2DM showed that trabecular BMD and cortical thickness are higher at the tibia and radius while cortical porosity is higher at the radius in T2DM subjects compared to controls [22]. No significant reduction in failure load as a measure of bone strength by fine element analysis at the radius and tibia was detected by the meta-analysis [22]. The elevation in cortical micro-pores is significantly associated with poor glycemic control and higher fracture risk [23]. Other factors contributing to impaired bone strength in T2DM are the accumulation of advanced glycation end products (AGEs), particularly pentosidine, insulin resistance, inflammatory cytokines, oxidative stress, and microvascular damage [23]. Emerging evidence suggests that serum AGEs concentration is inversely associated with indexes of cortical bone quality; additionally, AGEs content in the cortical, but not in the trabecular bone, is increased in T2DM [24,25]. A higher concentration of AGEs was observed in the collagen matrix of trabecular specimens collected during hip arthroplasty in T2DM subjects and associated with impaired bone strength [26]. In terms of fracture risk, data from meta-analyses showed an increased risk of incident vertebral and non-vertebral (hip, forearm) fractures in T2DM [27]. Diabetic patients are exposed to diffuse vascular damage. In this context, it has been suggested that decreased blood perfusion may contribute to the low bone density observed in diabetic patients. Moreover, reduced perfusion is likely coupled with impairment of the adequate response to the consequent hypoxia [28].

In patients with CKD, the pathophysiology of the skeleton is more complex, given the concomitant diabetic bone disorder and chronic kidney disease–mineral and bone disorder (CKD-MBD) [28,29]. The last is defined as a systemic disorder of bone and mineral metabolism associated with CKD and involving biochemical abnormalities, altered bone turnover, mineralization, volume, linear growth and strength, and vascular and soft tissue calcification, with increased risk for fractures, cardiovascular events, and mortality [28]. Main factors playing a central role in CKD-MBD are associated with the interplay between phosphate metabolism, parathyroid hormone (PTH), fibroblast growth factor 23 (FGF-23), and vitamin D [28]. A low or high bone turnover state may develop, with consequent perturbation of the bone quality [28]. In particular, a lower trabecular bone volume and thickness have been described in low-turnover states and a reduced mineral-to-matrix ratio and stiffness have been described in high-turnover states [30]. In terms of epidemiology, a higher prevalence of low-turnover disease (i.e., adynamic bone disease) was observed in the last decades in some cohorts, particularly among Caucasian subjects, and presumably in relation with diabetes itself, aging, the use of bone-active drugs, and increased patients survival [31].

Beyond the biochemical evaluation, clinical assessment of CKD-MBD includes the measurement of three-site (lumbar spine, femur, and forearm) BMD by dual X-ray absorptiometry (DXA), as recommended by the most recent guidelines, with a T-score ≤ −2.5 being predictive of fracture risk in CKD stages 3–5D, as well as in the general population [28,32].

The trabecular bone score is reduced in roughly half of patients with CKD stages G2-G5D [33,34]. The assessment of bone quality by HR-pQCT showed that many trabecular and cortical parameters may be altered in DKD [35]. Trabecular measures using HR-pQCT (density, BV/TV, number, and thickness) directly correlate with those obtained by bone biopsy and TBS, while radius cortical density using HR-pQCT correlates with histomorphometric parameters of bone remodeling [33]. 

In patients with CKD-MBD, perturbation of bone metabolism is associated also with osteomalacia, which may further increase fracture risk, as well as skeletal micro- and macro-vascular damage [29,36]. Fracture rates of 1.6–3% were reported in trials involving patients with DKD [29,37]. A recent post hoc analysis of the CREDENCE trial showed that the traditional osteoporotic risk factors (age, female gender, fracture history) were significant predictors of fracture in a population of 4397 patients with CKD [29]. Additionally, Asian ethnicity, low serum albumin, glycated hemoglobin, and a history of cardiovascular disease were significantly associated with fracture in the CREDENCE population, while factors related to CKD-MBD were not [29]. These data suggest that genetics, poor glycemic control, inflammation, excessive calcium load, and macrovascular complications may negatively affect bone health in DKD patients; the pathophysiology of skeletal damage and consequences on bone quality needs to be further addressed and characterized by clinical studies in this specific population.

Several studies have also connected diabetes, frailty, and malnutrition with an increased risk for osteoporosis and bone fracture [38]. The nutritional status also affects the capability of recovery following hip fracture [39]. The causality of this association is partially explained by the concomitant skeletal muscle atrophy, but also by other risk factors, including increased age, lower vitamin D levels, higher burden of comorbidities, and physical inactivity.

## 5. Antidiabetics and Bone Health in Patients with Normal Renal Function and in CKD

In addition to the disease itself, antidiabetic therapy may significantly impact bone metabolism. Different effects on bone health are exerted by various classes of antidiabetic drugs, as illustrated by experimental and clinical data (Table 1). The lack of agreement between studies in some cases, and the paucity of data from intervention studies specifically designed to address the issue, prevent the possibility of driving definite conclusions of the effects of antidiabetics on bone health. Notwithstanding, available evidence allows us to make reliable statements on the potential positive effects on bone mediated by metformin and glucagon-like peptide-1 receptor agonists (GLP-1RAs), neutral effects by dipeptidyl peptidase-4 inhibitors (DPP-4-i), sodium–glucose cotransporter-2 inhibitors (SGLT2i), and sulfonylureas (SUs), and negative effects by insulin and thiazolidinediones (TZDs) (Table 1). Recently, a large metanalysis on the risk of hip fracture with anti-diabetic drugs (metformin, SU, and insulin) has been published [40]. While metformin use was associated with a lower risk of hip fracture, the opposite was the case for insulin and SU. 

### 5.1. Experimental Studies

There are several experimental data on the effect of any antidiabetic class on bone in models with normal renal function; a fewer number of studies focused on CKD models. Table 1 summarizes the main evidence.

The action of metformin is mostly directed to stimulate bone formation processes through the expression of runt-related transcription factor 2 (RUNX-2), BMP-2, osteocalcin, and OSTERIX [41]. It favors the expression of OPG, thus inhibiting osteoclast differentiation mediated by the RANKL as well [41]. Additionally, metformin protects mesenchymal stem cell (MSC) damage and promotes their differentiation to the osteoblastic vs. the adipogenic lineage [41,42]. In a mouse model of obesity, histomorphometric and microcomputed tomography data demonstrated that metformin partially reverses the skeletal abnormalities, particularly reducing cortical bone resorption and bone marrow adipose tissue [42]. The more recent study by Duan et al. confirms the potential of metformin in promoting osteoblastogenesis while inhibiting the adipogenesis of MSC in a mouse model of T2DM; notwithstanding, the authors observed an increase in marrow adipose tissue in association with metformin administration, as well as in vitro induction of MSC apoptosis in conditions of high metformin concentration [43]. The hypothesis was made that a negative effect would result from a high dose of metformin on the bone marrow MSC and adipose tissue, as resembled in patients with intensive glucose control, in which there would be no benefit of metformin on bone health [40]. As such, the loss of the positive effect of metformin on bone with intensive control may also be related to hypoglycemic episodes and falls [12].

In rat models of CKD, metformin demonstrated to ameliorate renal function through its anti-inflammatory and antifibrotic effects mediated by the suppression of the expression of cytokines such as tumor necrosis factor (TNF)-α and β, interleukin (IL)-1β, activation of the adenosine monophosphate-activated protein kinase (AMPK), and decreased phosphorylation of extracellular-signal-regulated kinase 1/2 (ERK1/2) [44,45]. In models of CKD-MBD, metformin prevented an increase in serum creatinine, phosphate, PTH, and FGF23, and a decline in serum calcium in rats, with a consequent lower rate of high bone turnover disease and vascular calcification [45]. 

GLP-1RA may exert multiple effects on the skeleton [46]. In murine pre-osteoblasts, liraglutide activates osteoblastogenesis through the stimulation of the Wnt/β-catenin signaling via the phosphoinositide 3-kinase (PI3K)/protein kinase B (AKT), ERK1/2, and cAMP/protein kinase A (PKA) pathways [47]. As a consequence, GLP-1RA stimulates MSCs toward their differentiation to osteoblasts while reducing adipogenesis [48]. In rat models of T2DM, GLP-1RA treatment is associated with increases in the OPG/RANKL ratio, serum osteocalcin, and femoral BMD, stimulation of RUNX2 activity, and decreased expression of mRNA and serum levels of SOST/sclerostin [48]. Finally, amelioration of blood flow to bone was described in association with the exenatide treatment of diabetic mice as a possible mechanism of increased bone formation [46].

In animal models of DKD, GLP-1RA demonstrated nephroprotective effects through their diuretic, antioxidant, anti-inflammatory, and natriuretic actions [49]. Additionally, the administration of GLP-1RA was associated with reduction in glomerular sclerosis via the activation of AMPK and endothelial nitric oxide synthase (eNOS) with consequent decrease in urinary albumin; activation of autophagy through suppression of the mammalian target of rapamycin (mTOR) was described as well [49]. These actions of GLP-1RA were demonstrated in models of established and early CKD as effective in improving tubular-interstitial changes associated with diabetes [49].

Pre-clinical studies on the effects of DPP-4i on bone showed no consistent results [43]. Hypotheses on the possible actions of DPP-4i include indirect actions through the amelioration of serum glucose, enhancement of serum vitamin D levels, reduction in adipose tissue inflammation, and effects on energy metabolism, as well as differences in the effects of different molecules of this class [46,50,51]. In an ovariectomized mouse model of estrogen-deficient osteoporosis, Wang et al. demonstrated that sitagliptin reduces osteoclastic bone resorption by the inhibition of the downstream mechanisms of RANKL action [51]. Conversely, vildagliptin was associated with no significant effect on bone remodeling, while animal studies with saxagliptin reported a negative action of the molecule on RUNX2, osteocalcin, and collagen expression, as well as on mineralization [50]. Finally, DPP-4i modulate many interleukin and cytokine pathways, as well as T- and B-cell actions and macrophage actions involved in bone remodeling with different (negative in some cases, positive in others) effects on the skeleton [52]. 

Animal studies reported the expression of DPP-4 on proximal tubules, podocytes, and vascular smooth and mesangial cells [53]. The hypotheses of a direct and indirect (mediated by the reduction in GLP-1 degradation) stimulation of diuresis and natriuresis of DPP-4i at this level was made through the inhibition of the Na^+^/H+ exchanger isoform 3 (NHE3) [53]. Other mechanisms of DPP-4i action on the kidneys include their effect on the atrial and brain-derived natriuretic peptide (ANP and BNP), neuropeptide Y (NPY), peptide YY (PYY), stromal-cell-derived factor-1-α, oxidative stress, inflammation, and apoptosis, as illustrated in rat models of type 1 diabetes, T2DM, and hypertension [53]. The net results are a reduction in albuminuria and improvement in glomerulosclerosis, and tubular-interstitial damage associated with DKD [53].

Several mechanisms of SGLT-2i action on bone metabolism have been hypothesized, though not fully demonstrated [54]. Pre-clinical studies failed to show definite results; however, differences between molecules of this class were observed [6]. The induction of urinary glucose excretion and natriuresis with consequent relative hypovolemia may increase fall and fracture risk; hypermagnesemia, possible mild hypercalciuria, and an increase in phosphate reabsorption with secondary elevation in serum PTH levels may eventually stimulate bone resorption [54]. Finally, high FGF23 levels in response to hyperphosphatemia may reduce the conversion of vitamin D in its active form [54]. Studies in diabetic mice showed that canagliflozin administration may be associated with increases in bone resorption markers and the perturbation of trabecular and cortical microarchitecture; partial improvement in microarchitecture parameters were observed when canagliflozin was associated with insulin [55]. Preclinical studies with empagliflozin reported the potential of the drug to decrease the expression of RANKL and markers of inflammation while enhancing BMP2 expression [55].

Nephroprotective effects of SGLT2i rely on different mechanisms of action. Hemodynamic effects are exerted by a reduction in the intraglomerular pressure, as well as improvement in renal congestion mediated by movement of water from the interstitium to the urine [56,57]. Favorable effects of SGLT2i on the tubulointerstitial function were observed in animal models of kidney injury through a reduction in glucose concentration and oxidative stress in the proximal tubule [57]. Weight loss, reduction in serum glucose and insulin levels, and the natriuretic action of SGLT2i were linked in a rat model of obesity and hypertension with the anti-hypertensive effects of these drugs [57]. Finally, a reduction in acute kidney injury was observed in mouse and rat models in association with an increased tubular expression of vascular endothelial growth factor, reduced energy expenditure in the proximal tubule, and amelioration of tubular oxygenation [56,57].

Pre-clinical data on SU suggest the potential of glimepiride in stimulating bone formation. In ovariectomized rats, glimepiride could reverse bone resorption induced by estrogen deficiency while stimulating bone formation; the same study reported that bone formation may be stimulated to a lesser extent in non-ovariectomized rats [58]. Similar results were observed by Ma et al. in rat osteoblasts cultured with different concentrations of glucose; the activation of eNOS stimulated by glimepiride through the PI3K/AKT pathway may be associated with the induction of osteoblast differentiation [59].

Similarly to glimepiride, in vitro and in vivo studies demonstrated the anabolic effects of insulin on bone [60]. The administration of insulin in cultured osteoblasts showed to promote their proliferation and production of alkaline phosphatase (ALK) and collagen through a possible inactivation of cell apoptosis and mitogenic stimulation [61]. In animal models of insulin deficiency, impairment in mineralization, mechanical properties, and reduced cell proliferation and collagen synthesis were described; insulin administration may reverse these bone characteristics [61].

Several experimental studies investigated the effects of TZD on bone health. In the aggregate, they demonstrated that both pioglitazone and rosiglitazone increase bone resorption and bone marrow adiposity while inhibiting bone formation [62,63]. Acting on the peroxisome proliferator-activated receptor γ (PPAR-γ), TZD decreases MSC differentiation toward osteoblasts and increases the adipocyte number; RANKL expression is increased, as well, with the consequent stimulation of osteoclastogenesis [62,63].

### 5.2. Clinical Studies

There has been evidence of conflicting results between experimental and clinical studies on the skeletal effects of some classes of antidiabetic drugs (Table 1). 

Metformin demonstrated to have potential positive effects on BMD and bone properties. An 18-month randomized clinical trial (RCT) in a total of 407 patients treated with the association of metformin and insulin compared to placebo and insulin demonstrated that femoral neck BMD does not decline in the metformin group [64]. Similar results were reported by other studies [65,66,67]. Interestingly, in a recent retrospective study in a total of 11,458 patients with T2DM aged 40 and older in a single center in China, metformin use was associated with higher T-scores at the femur and lumbar spine regardless of age, BMI, and GFR [66]. In terms of bone properties, treatment with metformin was associated with a lower concentration of pentosidine in the cortical bone assessed by high-performance liquid chromatography on bone biopsy specimens in 25 postmenopausal women with T2DM [68]. There are no definitive results from systematic reviews and meta-analyses on the possible anti-fracture efficacy of metformin. Hidayat et al. reported a significant relative risk reduction in metformin users in a meta-analysis of 12 observational studies [69]. Similarly, an inverse relationship between metformin use and risk of fracture was described in a systematic review and meta-analysis of six observational studies [70]. A more recent network meta-analysis of 161 studies in a total of 191,361 patients treated with different classes of antidiabetics reported no influence of metformin on fracture risk [71]. 

Similarly to metformin, there is not an overall agreement between clinical studies in reporting benefits on the bone health of GLP-1RA, as well as significant antifracture efficacy. A recent systematic review on the effect of GLP-1RA on bone metabolism showed that exenatide and liraglutide had no significant effect on BMD, thus implying that the drugs may prevent BMD reduction associated with weight loss [48]. No significant changes in bone turnover markers (BTMs) were reported in all but one study, which showed an increase in the bone formation marker procollagen type I N-terminal propeptide (P1NP) [48]. As far as fracture risk is concerned, the meta-analysis by Cheng et al. including 38 RCTs with 39,795 patients with T2DM explored the effect of liraglutide or lisixenatide vs. placebo or other antidiabetic drugs [72]. The authors reported an overall significant reduction in fracture risk in GLP-1RA-treated patients [72]. Similar results are described by the more recent network meta-analysis by Tsai et al., where the use of GLP-1RA demonstrated higher antifracture efficacy compared to placebo and other classes of drugs [71]. Similar data are not available in patients with T2DM and CKD. Conversely, a meta-analysis of data in 8505 patients treated with GLP1-RA from four real-world studies reported no significant impact of these antidiabetics on fracture risk [73].

Clinical studies in patients treated with DPP-4i showed both neutral and positive effects on BMD and BMTs by this class of drug [74,75,76]. A retrospective analysis of 200 patients with T2DM reported a similar BMD increase after 12 months in the DPP-4i and in the control group (treated with other classes of antidiabetics), and a trend toward a higher TBS value in the DPP-4i group [76]. Several meta-analyses were performed to explore the association between DPP-4i and fracture risk, including a vast number of RCTs performed in thousands of patients with almost all molecules of this class and compared with placebo or other antidiabetics [71,77,78,79]. They collectively demonstrated that there is no significant association between DPP-4i use and fractures. A recent network meta-analysis of 177 RCTs in a total of 165,081 participants with a median follow-up of 26 weeks reported that DPP-4i do not increase fracture risk compared with placebo, insulin, metformin, SU, TZD, and alpha-glucosidase inhibitors [79]. The same conclusion was drawn by Driessen et al., who performed a meta-analysis of four real-world studies including 22,961 DPP4-i users and compared it with other antidiabetic drugs [73]. 

In CKD, Cowan et al. recently reported data from a population-based study in a total of 37,449 new DPP-4i users in Ontario, Canada aged 66 and older and followed up for 365 days [80]. In the subgroup analysis by estimated GFR category, the authors did not observe any increase in fracture incidence in moderate-to-severe CKD [80].

In human studies, SGLT2i demonstrated to exert various effects on BTMs depending on the molecule and the specific marker [54]. Indirect mechanisms of SGLT2i action on bone turnover were postulated, including the weight loss associated with their administration, but have not been fully clarified [54]. Administration of canagliflozin was associated with increases in both resorption [type I collagen carboxyl-terminal peptide β (β-CTX)] and formation (osteocalcin) markers, while no changes were seen in patients treated with dapagliflozin and empagliflozin [55,81,82]. Akin results were reported as far as BMD is concerned, canagliflozin being associated with BMD reduction, while other molecules were not [55,81,82]. A 104-week, placebo-controlled, phase three clinical trial of canagliflozin vs. placebo showed a 1.2% significant decrease in total hip BMD in the canagliflozin groups that was higher compared to placebo (−0.9%) [81]. No changes in BMD were observed at other skeletal sites, or in the bone quality parameters in association with canagliflozin administration [81]. Interestingly, a reduction in body weight explained only a small portion of the variability in serum β-CTX in this trial [81]. In this context, a retrospective cohort study in 34,960 adults with diabetes treated with SU or SGLT-2i in the UK concluded that fracture risk is not increased in SGLT-2i users even after stratification for BMI decrease [83].

Results from systematic reviews and meta-analyses are not consistent and definitive in delineating the fracture risk profile of SGLT-2i. Notwithstanding, a general conclusion of a substantial neutral effect on fracture risk of the SGLT-2i could be made. The negative effect on fracture observed in the Canagliflozin Cardiovascular Assessment Study (CANVAS) was not confirmed by other studies [71,84,85]. In a pooled analysis of CANVAS, Watts et al. demonstrated that the incidence of fracture was significantly higher in the canagliflozin (4%) group compared to the placebo (2.6%) group [84]. In particular, older age, higher cardiovascular risk, and lower GFR were associated with higher fracture incidence in the canagliflozin group, as well as a possible higher fall rate [84,86]. The analysis of the RCTs with canagliflozin not including the CANVAS excluded any difference in fracture incidence between canagliflozin- and non-canagliflozin-treated patients [81]. More recent network meta-analyses substantially confirmed the absence of any association between SGLT-2i administration and fracture risk [71,79].

In CKD, a number of studies demonstrated clinical benefits from the nephroprotective effects described by experimental studies with SGLT-2i. In particular, SGLT2i reduced the risk of dialysis, transplantation, acute kidney injury, and mortality associated with renal disease [56]. In terms of fracture risk, there have been reports of increased risk in patients with moderate renal impairment treated with canagliflozin and dapagliflozin [84,87]. Notwithstanding these results, the study by Cowan et al. failed to find any increase in fracture incidence in 38,994 new users of SGLT2i [80]. Additionally, a meta-analysis of 27 RCTs comparing SGLT2i to placebo in 20,895 participants with a mean follow up of 64 weeks showed that moderate-to-severe renal impairment is not a risk factor for fractures in patients with T2DM treated with different molecules of this class [85].

In patients treated with SU, there have been some inconstant reports of significant effects of these drugs on BTMs; a reduction in serum CTX after 12 months was described by some authors, while others reported no significant changes [88,89]. Similarly, increases in some bone formation markers (e.g., osteocalcin), and no changes in others (e.g., P1NP) were observed in different clinical studies [89,90]. No significant changes in BMD at all sites were reported by clinical studies in SU-treated patients, as well as a substantial neutral effect on fracture risk [71,89,90,91]. In this context, possible higher fracture risk in association with SU use in older (≥65 years) patients has been recently described, presumably in association with higher risk of hypoglycemia [92].

The anabolic effects of insulin described by pre-clinical studies seem to somehow be confirmed in clinical studies describing higher BMD in patients with T2DM and hyperinsulinemia [61]. Additionally, there have been reports of higher BMD values in T2DM patients on insulin compared to those on oral antidiabetics [93]. Conversely, there was no description of BTMs changes during insulin therapy [94]. More notably, systematic reviews and meta-analyses reported higher fracture risk in patients with T2DM treated with insulin, possibly in association with higher risk of falls, as well as many other complications of diabetes (visual impairment, neuropathy, etc.) [61,69,95].

Differently from what has been observed for other antidiabetics, there is a good correlation between experimental and clinical data describing detrimental effects of TZD on bone. Billington et al. performed a systematic review and meta-analysis of 20 RCTs involving 3743 participants treated with rosi- and pioglitazone for 3–24 months and evaluated their effects on BMD and BTMs [96]. The authors reported modest BMD reduction at the lumbar spine, total hip, and forearm during TZD administration; changes in BTMs were observed in some but not all studies, with high heterogeneity and no significant association with BMD loss [93]. Higher fracture risk is invariably described by meta-analyses in association with TZD use when compared to placebo and any other antidiabetics [69,71]. The recent analysis of the relative ranking probability of fracture made in the network meta-analysis by Tsai et al. demonstrated that TZDs represent the class of drug with the highest probability of causing fracture compared to placebo, metformin, GLP-1RA, DPP-4i, SGLT-2i, SU, and insulin [71].

**Table 1:** Summary of data from experimental and clinical studies and meta-analyses on the skeletal effects of various classes of drugs employed in the treatment of type 2 diabetes.

Drug Class	Experimental Studies	Clinical Studies	Meta-Analyses on Fracture Risk
Metformin	-Stimulation of RUNX-2, BMP-2, osteocalcin, OSTERIX, OPG, differentiation of MSC toward osteoblasts -CKD: prevents increase in Cr, P, PTH, FGF23, vascular calcification, and decline in Ca	-Potential positive effects on BMD and bone properties -CKD: potential positive effects on BMD	-Neutral (potential positive) effect
GLP-1RA	-Stimulation of Wnt/β-catenin signaling, RUNX-2, osteocalcin, OPG, differentiation of MSC toward osteoblasts, increase in femoral BMD and blood flow -CKD: renoprotective effects	-No effect on BTMs and BMD (may prevent weight-loss-associated BMD reduction) -CKD: renoprotective effects, lower all-cause mortality	-Potential positive effect
DDP-4i	-Reduction in RANKL action (sitagliptin), RUNX2, osteocalcin, collagen, mineralization (saxagliptin); different effects through IL, cytokines, T- and B-cells -CKD: renoprotective effects	-Neutral and positive effects on BMTs and BMD; increase in TBS	-Neutral effect -CKD: neutral effect
SGLT-2i	-Increase in bone resorption markers, perturbation of microarchitecture (canagliflozin); stimulation of RANKL, inflammation, BMP2 (empagliflozin), -CKD: renoprotective effects	-Increase in β-CTX and osteocalcin, BMD reduction (canagliflozin); no effects on BTMs and BMD (other molecules); no effects on bone quality -CKD: reduced risk of dialysis, transplantation, AKI, and mortality	-Neutral effect -CKD: neutral effect
SU	-Stimulation of bone formation (glimepiride) through eNOS and PI3K/AKT	-Inconsistent data on effects on BTMs, neutral effects on BMD	-Neutral effect
Insulin	-Promotes osteoblasts proliferation, ALK and collagen production	-No effect on BTMs, increase in BMD	-Negative effect
TZD	-Stimulation of RANKL, MSC differentiation toward osteoblasts adipocytes through PPAR-γ	-Possible negative effects on BTMs, decrease in BMD	-Negative effect

RUNX-2, runt-related transcription factor 2; BMP-2, bone morphogenetic protein 2; OPG, osteoprotegerin; MSC, mesenchymal stem cells; Cr, creatinine; P, phosphate; PTH, parathyroid hormone; Ca, calcium; GLP-1RAs, glucagon-like peptide-1 receptor agonists; DDP-4i, dipeptidyl peptidase-4 inhibitors; SGLT-2i, sodium–glucose cotransporter-2 inhibitors; SUs, sulfonylureas; eNOS, endothelial nitric oxide synthase; PI3K/AKT, phosphoinositide 3-kinase/protein kinase B; ALK, alkaline phosphatase; TZDs, thiazolidinediones; PPAR-γ, proliferator-activated receptor γ; BMD, bone mineral density; BTMs, bone turnover markers; TBS, trabecular bone score; β-CTX, type I collagen carboxyl-terminal peptide β; AKI, acute kidney injury.

## 6. The Use of Antidiabetics in T2DM with and without CKD: From Standards of Care to Strategies for Supporting Bone Health

**Figure 1:**
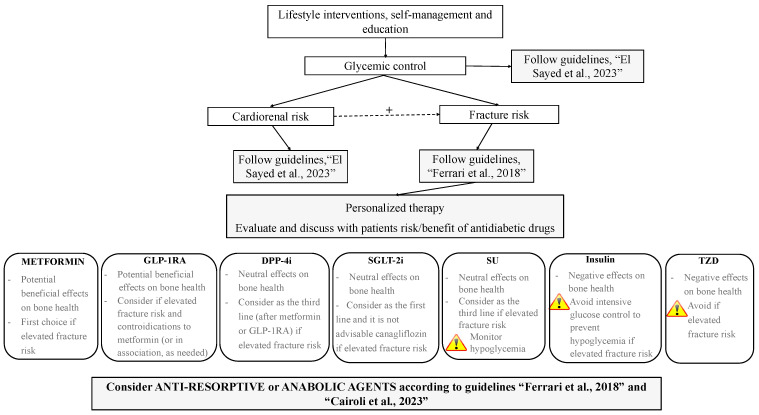
Proposed integrated approach to the management of antidiabetic therapy aimed at targeting fracture risk in patients with T2DM; +, positive influence; GLP-1RAs, glucagon-like peptide-1 receptor agonists; DDP-4i, dipeptidyl peptidase-4 inhibitors; SGLT-2i, sodium–glucose cotransporter-2 inhibitors; SUs, sulfonylureas; TZDs, thiazolidinediones. Guidelines: “El Sayed et al., 2023” [1], “Ferrari et al., 2018”, [2], “Cairoli et al., 2023” [97].

**Figure 2:**
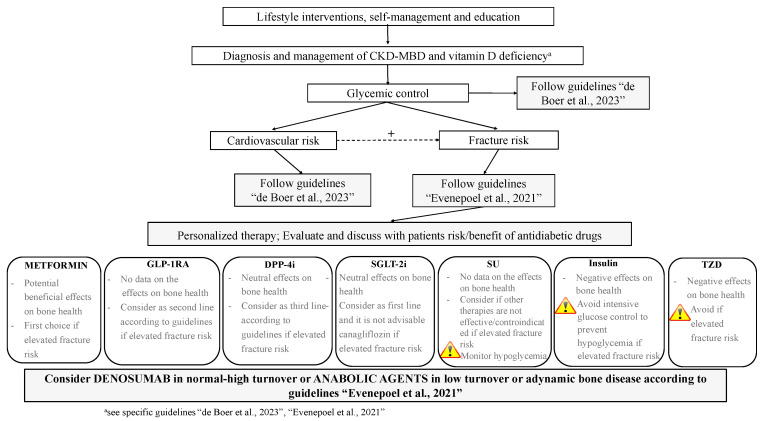
Proposed integrated approach to the management of antidiabetic therapy aimed at targeting fracture risk in patients with T2DM and CKD G4-G5D; +, positive influence; GLP-1RAs, glucagon-like peptide-1 receptor agonists; DDP-4i, dipeptidyl peptidase-4 inhibitors; SGLT-2i, sodium–glucose cotransporter-2 inhibitors; SUs, sulfonylureas; TZDs, thiazolidinediones. Guidelines: “de Boer et al., 2023” [12], “Evenepoel et al., 2021” [98].

As per the most recent ‘Standards of care in diabetes’, education promoting behavioral interventions on weight control and physical activity, self-management, and support are the first glucose-lowering strategies to be pursued in all diabetic subjects [1]. Pharmacological intervention should be considered in a holistic and patient-centered clinical approach aimed at targeting glucose control, management of body weight, and reducing the cardiorenal risk, as well as risk for side effects [1]. With this aim, risk stratification for cardiovascular disease, heart failure, and CKD is prompted in all patients, and combination therapy is recommended in the early stages as needed [1]. From the perspective of a real and effective integrated approach to diabetic patients, strategies aimed at best managing antidiabetic therapy including, among the recommendations, the fracture risk profile, cannot be overlooked. We therefore propose integrated approaches to the management of antidiabetic therapy in T2DM patients with and without CKD (Figure 1 and Figure 2). 

Lifestyle interventions, self-management, and education need to be pursued in all patients (Figure 1) with the aim of facilitating knowledge on the skeletal complications of T2DM, assessing fracture risk (according to guidelines [2]) and promoting physical activity to improve postural stability, reduce risk of fall, and ameliorate BMD and bone quality [95]. Pre-clinical and clinical studies demonstrated the beneficial effects of exercise training on bone metabolism in rat models and in patients with T2DM [99,100]. In particular, increases in bone turnover in association with improvement in glycemic control and decreased body weight and fat mass were observed in diabetic patients, as well as the maintenance of BMD regardless of lower body weight even in older adults [101]. The annual evaluation of falls and interventions aimed at reducing risk of falls (exercise, management of medication also avoiding hypoglycemic episodes, vision assessment, etc.) should be employed, particularly in the elderly [100]. Assessment of fracture risk is desirable in all patients with T2DM, as well as the evaluation of risk for cardiovascular and renal disease. Indeed, the close relationship existing between cardiovascular and renal disease and skeletal fragility makes interventions aimed at modifying cardiorenal risk beneficial for bone health as well [98]. Similarly, patients need to be encouraged to achieve and maintain glycemic control with the aim of also reducing fracture risk. As is well known, there is a significant relationship between glycemic status and skeletal fragility in diabetes, with any 1% HbA1c increase being associated with an 8% higher fracture risk [102]. Hence, the personalization of antidiabetic therapy should be discussed with patients based on risk/benefits of fracture risk assessment, as well as the drug-associated risk of hypoglycemia and falls. With this aim, the use of TZD should be avoided (or discontinued if ongoing) if fracture risk is elevated. Metformin should be considered as first choice, according to comorbidities, as potentially effective in reducing fracture risk. Alternatively, or in combination with metformin, GLP-1RA should be considered as a good choice in patients with skeletal fragility. Owing to substantial neutral effects on bone, DPP-4i and SGLT-2i may be considered as a third line in high-risk patients; given the lack of homogeneity among studies, differences among various molecules of the class of the SGLT-2i should be made and canagliflozin should be avoided. As far as SU and insulin are concerned, the risk of hypoglycemia should be carefully monitored in patients treated with these drugs, particularly in the elderly, in relation to a higher risk of falls and fractures [95]. As starting insulin is commonly unavoidable, we suggest this drug to be employed as late as possible when fracture risk is elevated. Finally, a bone-active agent should be started according to clinical indications and recommendations [97].

As in non-CKD patients, lifestyle intervention targeting traditional risk factors (i.e., increasing the level of physical activity, smoking cessation, reducing alcohol intake and risk of fall) is warranted in CKD patients G4-G5D (Figure 2) [12]. Before considering specific treatments, it is mandatory to diagnose the specific disorder in the setting of CKD-MBD, manage vitamin D deficiency, and avoid hypercalcemia, hyperphosphatemia, and excessive calcium load if present and/or overtreatment with antiparathyroid agents [12]. A routine evaluation of fracture risk by means of BMD measurement and the use of a specific tool (i.e., FRAX^®^) has predictive value for incident fractures in the CKD population [98]. Circulating levels of calcium, phosphate, PTH, 25-hydroxy-vitamin D, and bone-specific ALK can be used to evaluate CKD-MBD; markedly high or low levels may indeed reflect the underlying bone turnover; bone biopsy could be performed when the diagnosis is not clear [28]. Even though few data on the skeletal effects of antidiabetics in CKD G4-G5D patients are available, suggestions on their management can be made on the basis of the current evidence. Guidelines on the management of T2DM in CKD should be followed, preferring the use of metformin and SGLT-2i other than canagliflozin as appropriate [12]. GLP-1RA and DDP-4i should be considered as second/third line therapy, as few data are available on their effects on fracture risk in CKD. SU and TZD should be avoided in the majority and in all patients, respectively, as no data on skeletal effects in CKD are available for the first class and negative effects are described for the second class. Finally, the risk of hypoglycemia should be avoided, especially when therapy is implemented and insulin therapy is started.

Pharmacologic strategies that inhibit bone resorption (mostly denosumab in CKD G4-G5D, as it does not own any renal excretion) may be helpful in preventing bone loss and fracture in patients with normal- to high-turnover bone disease, while anabolic agents may be employed in patients with low turnover (i.e., adynamic bone disease).

## 7. Perspectives

Skeletal fragility in diabetic patients with or without CKD is a common finding that physicians of different specialties need to face in routine clinical practice. In the last decade, there have been essential changes in the management of T2DM toward the definition of personalized therapy in terms of the achievement of effective glucose control and preventing comorbidities. Although complex, the inclusion of a skeletal fragility profile and implementation of strategies aimed at reducing fracture risk in managing antidiabetic therapy is needed in the era of personalized and patient-centered therapy. Large-scale RCTs including the skeletal effects of anti-diabetic medications as the primary endpoint are definitely warranted in both CKD and non-CKD patients to define a practical and evidence-based algorithm for the holistic approach to these patients.

## Data Availability

Not applicable.
